# Myeloid Cells as Clinical Biomarkers for Immune Checkpoint Blockade

**DOI:** 10.3389/fimmu.2020.01590

**Published:** 2020-07-24

**Authors:** Elisa Peranzoni, Vincenzo Ingangi, Elena Masetto, Laura Pinton, Ilaria Marigo

**Affiliations:** ^1^Center for Therapeutic Innovation in Oncology, Institut de Recherche International Servier, Suresnes, France; ^2^Veneto Institute of Oncology IOV - IRCCS, Padua, Italy

**Keywords:** myeloid cells, predictive biomarkers, MDSC (myeloid-derived suppressor cell), TAM (tumor-associated macrophage), circulating biomarkers, resistance to immunotherapy, immune checkpoint inhibitors, tumor biomarkers

## Abstract

Immune checkpoint inhibitors are becoming standard treatments in several cancer types, profoundly changing the prognosis of a fraction of patients. Currently, many efforts are being made to predict responders and to understand how to overcome resistance in non-responders. Given the crucial role of myeloid cells as modulators of T effector cell function in tumors, it is essential to understand their impact on the clinical outcome of immune checkpoint blockade and on the mechanisms of immune evasion. In this review we focus on the existing clinical evidence of the relation between the presence of myeloid cell subsets and the response to anti-PD(L)1 and anti-CTLA-4 treatment. We highlight how circulating and tumor-infiltrating myeloid populations can be used as predictive biomarkers for immune checkpoint inhibitors in different human cancers, both at baseline and on treatment. Moreover, we propose to follow the dynamics of myeloid cells during immunotherapy as pharmacodynamic biomarkers. Finally, we provide an overview of the current strategies tested in the clinic that use myeloid cell targeting together with immune checkpoint blockade with the aim of uncovering the most promising approaches for effective combinations.

## Introduction

Immune checkpoint inhibitors (ICIs) have proven their efficacy in boosting the effector functions of tumor-reactive T lymphocytes against cancer cells. ICI activity is carried out through the specific targeting of negative immune checkpoint molecules or their ligands, expressed on either T cells or myeloid and tumor cells (1). Since 2011, the FDA has approved 7 ICIs (one anti-CTLA-4, three anti-PD-1, and three anti-PD-L1 antibodies) for several indications and many more drugs are in preclinical and clinical development. However, despite the exponential increase in the use of ICIs in the clinic, most patients with advanced cancers still do not respond to these treatments.

It is therefore imperative to understand the mechanisms of action of these drugs to better select responder patients before or during treatment, as well as to design new drugs or combinations that could increase the chances of clinical response and, at the same time, limit the exposure to adverse effects and ineffective therapies for non-responding patients. The importance of reliable biomarkers is progressively recognized for successful clinical trials and for the comprehension of ICI. Most of the understanding for the approved immune checkpoint blockers comes from preclinical experiments and still few clinically validated biomarkers are available.

Not surprisingly, biomarkers are currently mainly focused on T cells and tumor cells, but it is becoming clear that other cell types in the periphery and at the tumor site can impact the efficacy of immunotherapy. Myeloid cells are among the “usual suspects,” given their plasticity and their well-known role as immune modulators in tumor growth and metastasis (2–5). The modulation of ICI response by cells of the myeloid lineage is currently being examined, mainly at the preclinical level. Exploratory biomarkers in recent clinical trials have confirmed the necessity to take into account the presence of these cells for the selection of patients that could benefit from immune checkpoint blockade and the design of ICI combinations with myeloid-targeting agents (6–8).

**Figure 1:**
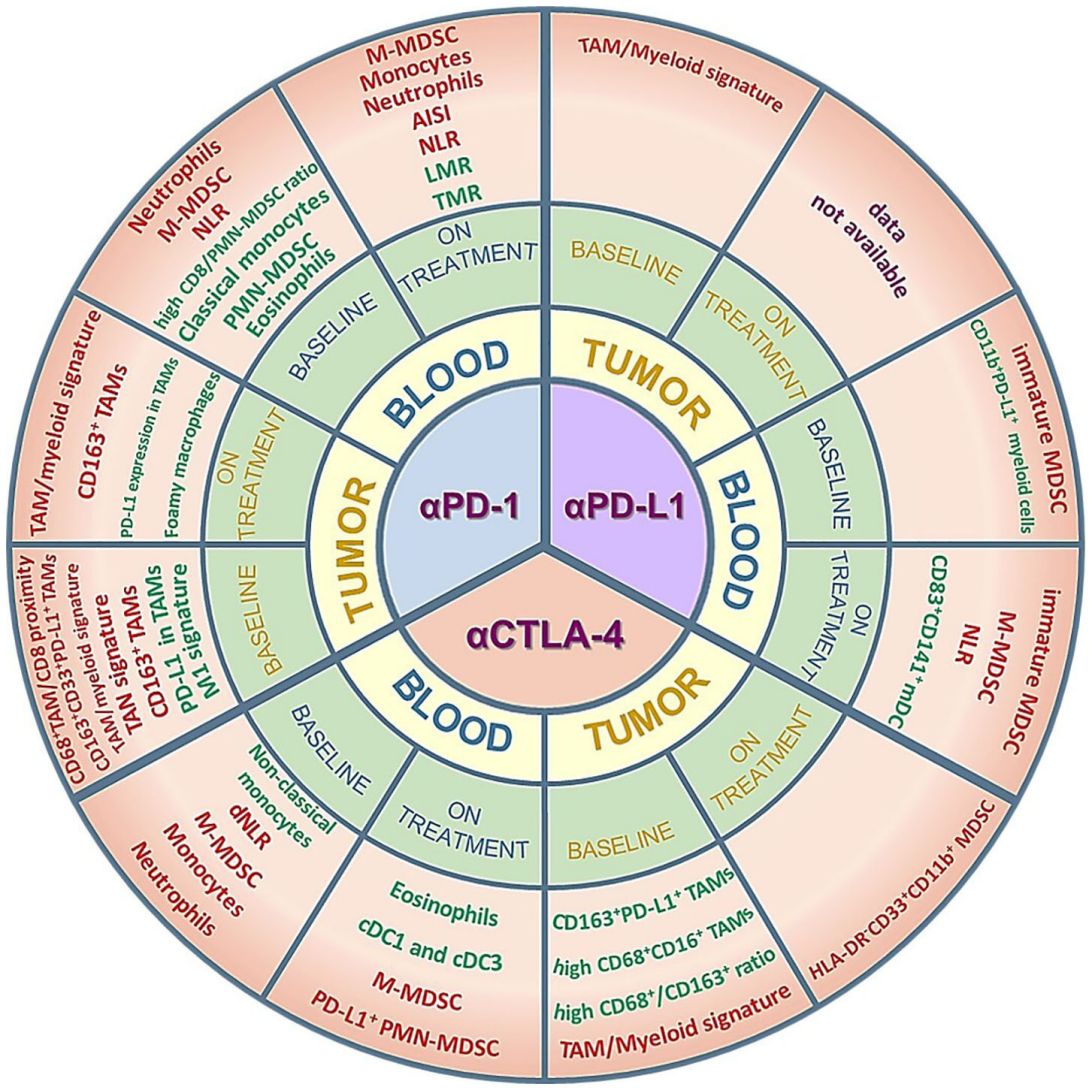
Myeloid cell subsets as potential predictive biomarkers in ICI-treated patients. The figure summarizes the clinical data on circulating or tumor-infiltrating myeloid cells that are described as predictive of response/improved survival (green) or resistance/worse survival (red) in cohorts of patients treated with anti-PD-1, anti-PD-L1 or anti-CTLA-4 antibodies. *Positive predictors* (green) are myeloid subsets whose amounts are either higher than a specific cut-off value and associated to response/improved survival or lower than a specific cut-off value and associated to resistance/worse survival. Conversely, *negative predictors* (red) are myeloid subsets whose amounts are either higher than a specific cut-off value and associated to resistance/worse survival or lower than a specific cut-off value and associated to response/improved survival. The myeloid subsets are described in more detail in the main text and in the Supplementary Table 1. AISI, aggregate index of systemic inflammation = platelet count x AMC x NLR; NLR, neutrophil-to-lymphocyte ratio; dNLR, derived neutrophil-to-lymphocyte ratio; LMR, lymphocyte-to-monocyte ratio; TMR, Tregs to Lox-1+ PMN-MDSCs ratio; TAM, tumor-associated macrophages; TAN, tumor-associated neutrophils; M- or PMN-MDSC, monocytic- or polymorphonuclear-myeloid-derived suppressor cells; mDC, myeloid dendritic cells; cDC, conventional dendritic cells.

Given the challenging translation of preclinical results into the clinical setting, especially for the phenotypic description of cell subsets, in this review we to focus on the clinical evidence of the predictive value of myeloid cells, both at baseline and during treatment, in response to the approved ICIs. An overview of the myeloid biomarkers that will be described can be found in Figure 1 and Supplementary Table 1. In addition, we report some promising clinical results of ICI combinations with myeloid-targeting drugs, highlighting the importance of modulating these cell players for successful immunotherapy.

## Biomarkers in ICI Therapy

Biomarkers are molecular or cellular parameters, measured in fluids and tissues, that give information about the disease, the condition of the host, the prognosis and the response to a treatment. In the context of a clinical trial, several types of biomarkers can be studied: *prognostic biomarkers*, that give information about the outcome of patients irrespective of the treatment, *predictive biomarkers*, that estimate whether an individual is likely to respond to a specific treatment, *pharmacodynamic biomarkers*, that evaluate the impact of a drug on its target and on disease progression, and *safety biomarkers*, that can rapidly alert on the toxicity of a therapy (9–11).

In this review we chose to focus on potential predictive and pharmacodynamic biomarkers, given their relevance for translational medicine. Predictive biomarkers can be measured at baseline or during treatment, helping in the selection of patients that can most benefit from a treatment or in the rapid adaptation of the therapy, respectively. This rational selection reduces the risk of exposing non-responding patients to adverse effects. Pharmacodynamic biomarkers allow to understand if patients are responding to the administered drug and to shed light on the mechanism of action of ICIs and their impact on the tumor microenvironment (TME) and the immune system. They thus help in a rapid assessment of response and can guide the choice of combinations.

However, as some authors have pointed out, the formal distinction between predictive and prognostic biomarkers requires randomized trials with two treatment arms, where the effect of a biomarker is evaluated in both the treatment and the control group (12). As many of the studies reviewed here comprise early phase clinical trials with only one arm of treated patients, we cannot exclude the possibility that the described predictive biomarkers might be instead prognostic or both predictive and prognostic. We thus propose to consider them as potentially predictive, unless otherwise specified, but we also recommend to formally confirm the predictive value of these biomarkers in ICI therapy through two-arm randomized trials.

Biomarkers can be broadly divided into *circulating* (non-invasive and measurable in the blood) and *tumor* biomarkers. Since immunotherapy can be accompanied by significant toxicities, high costs and the complexity of obtaining biopsies, the development of complementary approaches, like non-invasive biomarkers, is fundamental to maximize the therapeutic efficacy and the success of clinical trials. *Circulating biomarkers* ensure a finer follow-up of patients at baseline, during and after treatment, permitting the early detection of relapse or resistance and the rapid adjustment of therapy (13, 14). Different biomarkers, such as circulating tumor DNA, circulating tumor cells, cytokines, exosomes and factors such as lactate dehydrogenase (LDH) and C-reactive protein (CRP) can be analyzed using liquid biopsies (15–17). Additionally, investigating the presence and the dynamics of peripheral blood leukocytes may unveil important predictive and pharmacodynamic biomarkers.

In the context of ICIs, there are no validated circulating predictive biomarkers yet. Nonetheless, blood tumor mutational burden (bTMB) is gaining interest because it shows a good correlation with TMB in non-small cell lung cancer (NSCLC) and has thus the potential to become a useful non-invasive predictive biomarker (18). Regarding pharmacodynamic markers, several authors have observed an increase in Ki-67^+^PD-1^+^ T cells, representative of a reinvigoration of exhausted lymphocytes, as well as an expansion of tumor-specific T cell clones, in the circulation of responders to ICIs (19–21).

However, circulating cell subsets other than T lymphocytes might also be relevant in immunotherapy. In this regard, the accumulation of myeloid-derived suppressor cells (MDSCs) has been proven to impair the efficacy of anti-tumor therapies in human cancers (22).

MDSCs are cells of myeloid origin with systemic expansion in cancer that can be distinguished from mature, terminally differentiated myeloid cells for their phenotype and for their immune-suppressive functions. Before the definition of standards for their identification in humans by a group of experts in the field (23), many partially overlapping subsets had been described, leading to confusion in the investigation of their biological role. Three main categories of human MDSCs exist: polymorphonuclear-MDSC (PMN-MDSC, Lin^−^CD11b^+^CD15^+^CD14^−^), monocytic MDSC (M-MDSC, Lin^−^CD11b^+^CD14^+^HLA-DR^low^) and early-stage MDSC (eMDSC, Lin^−^CD11b^+^CD33^+^CD14^−^CD15^−^HLA-DR^−^), each containing different subsets with peculiar biochemical and molecular markers. Besides the phenotypic characterization, the gold standard for MDSC definition remains however their immunosuppressive activity (23). Since these cells play a pivotal role in the establishment of a potent immunosuppression, both at a systemic and at the tumor level, some studies have started to explore their potential as biomarkers of response to ICIs (6–8).

Besides MDSCs, the modulation of the expansion and function of monocyte subsets has also been demonstrated to have a role in different diseases (2, 24). Human monocytes can be quantified by Coulter Counter impedance technology through the absolute monocyte count (AMC) (25) or by multi-color flow cytometry. Three major populations can be discerned based on CD14 and CD16 expression: classical (CD14^+^CD16^−^), non-classical (CD14^dim^CD16^+^), and intermediate (CD14^+^CD16^+^) monocytes, with distinct surface markers and functions. Classical monocytes exhibit a pro-inflammatory phenotype and are mainly involved in anti-microbial responses, adhesion to the endothelium, migration, and phagocytosis Intermediate and non-classical monocytes emerge sequentially from the pool of classical monocytes: intermediate monocytes are specialized in antigen presentation and transendothelial migration, while non-classical monocytes are responsible for complement and FcR-mediated phagocytosis, transendothelial migration and anti-viral responses (24, 26).

In addition to MDSCs and monocytes, the systemic expansion of neutrophils, eosinophils and immature myeloid cells has also been shown to reflect the immunosuppressive status of TME during immunotherapy (27). Neutrophil quantification can be done with Coulter counters through the absolute neutrophil count (ANC) or by flow cytometry (CD15^+^CD16^+^ cells). The neutrophil-to-lymphocyte ratio (NLR), calculated as the ratio between the ANC and the absolute lymphocyte count (ALC), is also used to illustrate the expansion of the neutrophil component to the detriment of lymphocytes, unveiling the patient inflammatory status, while its derivative form, dNLR, is given by the following formula: ANC/(WBC-ALC), where WBC is the total number of white blood cells.

Besides blood markers, investigators have also analyzed the tumor in search for predictive biomarkers. With an increasing number of ICI-treated patients and the intensified use of multiparametric analyses, new biomarkers are continuously discovered. For the approved anti-PD-1 blockers, several *tumor biomarkers* have been proposed, with variable level of clinical validation on large cohorts of patients and different cancers: PD-L1 staining (on tumor cells or both tumor and immune cells) evaluated by immunohistochemistry IHC, TMB, and the microsatellite instability (MSI) status, the infiltration of CD8 T cells, some transcriptomic signatures as TIS (Tumor Inflammation Signature) and TIDE (Tumor Immune Dysfunction and Exclusion), the presence of B cells and tertiary lymphoid structures, as reported elsewhere (9–11, 28–30). However, the predictive power of these biomarkers varies across tumors and it seems important to combine at least some of them to better distinguish potential responders from non-responders.

Even though the first markers were linked to features of tumor cells and effector T cells, the growing interest in profiling the whole TME is unveiling the potential predictive value for immunotherapy of tumor-infiltrating myeloid cells. The main myeloid populations found in tumors are tumor-associated neutrophils (TANs), dendritic cells (DCs), tumor-associated macrophages (TAMs), and monocytes (31). Depending on the TME, these myeloid populations can adopt very different phenotypes with distinct impact on the anti-tumor immune response, angiogenesis and invasion. Many reviews have analyzed the influence of tumor-infiltrating myeloid cells on the prognosis of cancer patients (32–35) and the mechanisms of negative and positive regulation of the anti-tumor immune response (3, 4, 36). Although most findings come from murine tumors, some clinical data are emerging that link the presence of myeloid cells in the TME with the outcome of approved immune checkpoint therapies, as discussed later (6–8).

## Anti-PD-1 Inhibitors

The immune-checkpoint Programmed cell Death protein 1 (PD-1) is physiologically up-regulated following lymphocyte activation, and, through the direct interaction with its ligands PD-L1 and PD-L2, limits the activity of T lymphocytes to prevent excessive immune responses (37–39). The anti-PD-1 monoclonal antibodies (mAb) Nivolumab and Pembrolizumab block the interaction of tumor-reactive T cells expressing PD-1 with leukocytes and tumor cells expressing PD-L1 and PD-L2 (40). These antibodies are approved for the treatment of several cancer types, in monotherapy or combination with chemotherapy and anti-angiogenic drugs. Despite these antibodies have improved the clinical outcome in a wide range of tumors, the long-term benefits are restricted to a small proportion of patients, emphasizing the need for more reliable biomarkers and new drug combinations (40–45).

The evaluation of PD-L1 expression in tumors by IHC, TMB, and MSI status at baseline are the only biomarkers used in the clinical practice with ICIs but none of them alone is a strong and universal predictor of response. As an example, PD-L1 staining shows intra-patient tumor heterogeneity, evidence of response to ICIs in patients with low/negative PD-L1 expression and a lack of technical standardization (9–11, 13, 46).

Together with baseline biomarkers, on-treatment evaluation of tumors can give important insights into the mechanisms of action and resistance of ICIs, helping clinicians rapidly refine the therapy in a personalized way. Responder biopsies are characterized by an infiltration of T cells, especially CD8^+^, the upregulation of genes related to T cell recruitment, activation, proliferation, and cytotoxicity (e.g., *CXCL9, CXCL10, PDCD1, MKI67, GZMB, IFNG*, and IFNγ-regulated genes) and an augmentation of PD-L1 expression, as a consequence of IFNγ release in the TME (47, 48). As the modulation of the TME by ICIs is still unclear, especially for non-T cells, we highlight here the studies that suggest the use of circulating and tumor myeloid cells, at baseline or during treatment, as novel predictive or pharmacodynamic biomarkers for anti-PD-1 inhibitors.

### Circulating Biomarkers

#### Monocyte Lineage

Several reports indicate the baseline presence of the circulating M-MDSCs as potentially related to the response to anti-PD-1 blockade. As an example, advanced melanoma patients with fewer M-MDSCs among peripheral blood mononuclear cells (PBMCs) before Nivolumab treatment more likely belonged to the responder and stable disease groups, thus suggesting that long-term responses might be seen after Nivolumab even in patients that have failed prior immunotherapy (49). This concept was recently reinforced by the observation that an early accumulation of M-MDSCs expressing the immunomodulatory molecule galectin-9, associated with the concomitant expression of Tim-3 on lymphocytes, was related to primary and secondary resistance to Nivolumab in metastatic NSCLC. Compared to healthy volunteers, a statistically significant increase in CD14^−^CD15-HLA-DR^+^ dendritic cells and M-MDSCs (defined here as CD14^+^HLA-DR^+^CD33^+^), together with a reduced number of granulocytes, was found in these patients. Two weeks after Nivolumab administration a rapid decrease in the M-MDSC was observed in responders and in patients with stable disease, while the number remained constant in non-responders. In addition, the authors showed that the combined expression of Tim-3 on CD8^+^ T cells and galectin-9 on M-MDSCs impaired the secretion of IFNγ by activated CD8^+^ T cells in the presence of anti-PD-1 *in vitro*, further suggesting that M-MDSCs can confer resistance to Nivolumab treatment (50).

M-MDSC expansion was similarly associated with poorer response in a cohort of advanced melanoma patients treated with a combination of Nivolumab and a multi-peptide vaccine. In these patients, the presence of different suppressive populations, including an M-MDSC subset (defined as CD11b^+^HLA-DR^low^CD14^+^), a population of PMN-MDSCs (Lin^−^CD14^−^CD11b^+^CD15^+^) and regulatory T cells (CD3^+^CD4^+^CD127^low^FoxP3^+^), was assessed at baseline and during treatment. The authors showed a trend toward lower baseline Tregs and M-MDSC levels in non-relapsing patients as compared with relapsing ones, thus suggesting the negative impact of these circulating populations on the clinical outcome, without clearly distinguishing their predictive vs. prognostic value (51).

In a cohort of metastatic urothelial carcinoma (mUC) patients treated with Pembrolizumab, successive doses of anti-PD-1 decreased the frequency of PD-1^+^ M-MDSCs and eMDSCs, even if these changes were not statistically significant predictors of response. This decrease may indicate that MDSCs can downregulate immune checkpoints at their surface as a mechanism of resistance to ICIs, but it may also be the mirror of a positive immunotherapeutic response with reduction of immunosuppressive populations (52).

The accumulation of monocytes is also sometimes associated with a worse outcome in different tumor types, both at baseline and during anti-PD-1 therapy. A decrease in CD11c^+^CD14^+^CD16^+^HLA-DR^hi^ monocytes, accompanied by a significant increase in overall survival (OS), has been observed in recurrent glioblastoma after post-surgery Pembrolizumab, but only in patients that had also received neo-adjuvant anti-PD-1 immunotherapy before surgery (53). Chasseuil et al. reported a statistically significant decrease in OS in relation to an increase in the total monocytic fraction in pre-treatment blood samples from advanced melanoma patients treated with Nivolumab, suggesting its potential as prognostic biomarker (54). In addition, in a cohort of NSCLC patients, the post-treatment AMC was higher in non-responders compared to responder patients, suggesting a predictive role of monocytes in anti-PD-1 therapy (27). Interestingly, Sekine et al. also identified the increase in the lymphocyte-to-monocyte ratio (LMR) after the start of Nivolumab treatment as a good predictor of response for NSCLC patients (55). An explanation to the negative impact of the cells of the monocytic lineage on ICI response may rely on the variety of mechanisms by which these cells can alter T cell effector functions, including nutrient depletion, generation of reactive oxygen species (ROS) and up-regulation of immune checkpoint molecules (36, 56–58).

A high monocyte frequency is however not always associated with a poorer response, as demonstrated by Krieg et al. who found a higher frequency of classical monocytes (CD14^+^CD16^−^CD33^hi^HLA-DR^hi^), measured before therapy by single-cell mass cytometry, in melanoma patients responding to anti-PD-1. A flow cytometry validation confirmed that a frequency of classical monocytes higher than 19.38% before therapy was associated with a better treatment outcome. These conflicting results might be explained by the fact that these monocytes have higher amounts of migration and activation markers, such as ICAM-1 and HLA-DR, and might thus be actively involved in the anti-tumor immune response induced by anti-PD-1 (59). Indeed, classical monocytes express high levels of chemokine receptors to migrate to inflamed tissues and secrete pro-inflammatory soluble factors, potentially shaping inflammation, and being a key player in the anti-tumor response (24).

Similarly, in a cohort of advanced NSCLC patients, an AMC higher than 700/mm^3^ (median AMC for responders) at baseline was related to a shorter TTR (time to response) to either Nivolumab or Pembrolizumab. According to the authors, the positive role of monocytes could reflect a more intense macrophage-mediated tumor cell cytotoxicity that synergizes with the activity of tumor-reactive T lymphocytes. However, the impact of AMC on response to ICIs is likely highly specific to the tumor type, given that different tumors may release specific cytokines promoting the polarization of TAMs toward either an immunosuppressive or an anti-tumor phenotype (60).

#### Granulocyte Lineage

In a longitudinal study performed on the blood of NSCLC patients before and after Nivolumab treatment, the ratio of Tregs to PMN-MDSCs, TMR, was chosen as a predictor of response to treatment: a TMR ≥ 0.39 after the first infusion was associated with a higher probability of being a responder. This suggests that PMN-MDSCs, distinguished from normal neutrophils by the expression of the lectin-type oxidized LDL receptor-1 (Lox-1) in this study, could impair the efficacy of anti-PD-1 therapy, while highlighting at the same time an association between a higher frequency of Tregs and a better response to treatment. In these patients, CXCL2, CCL23, CX3CL1, and HMGB1 levels, known to be related to MDSC recruitment and proliferation, were also significantly higher in non-responders (61).

However, the role of PMN-MDSCs in tumors is still debated: in fact, they have also been proven to be associated with a better response to Nivolumab in advanced NSCLC patients. In this cohort, high baseline levels of PMN-MDSCs (Lin^−^HLA-DR^low/neg^CD33^+^CD13^+^CD11b^+^CD15^+^CD14^−^) and low baseline CD8/PMN-MDSC ratios were associated with a better OS. As a further confirmation of this results, researchers identified an immunological asset as a possible prognostic biomarker of OS and progression-free survival (PFS) after Nivolumab treatment: in a multivariate analysis, the combination of NLR <3, baseline levels of PMN-MDSCs ≥ 6 cell/μl, eosinophil count ≥ 90 cells/mm^3^ and neutrophil count <5,840 cells/mm^3^ showed a statistically significant association with good prognosis (62).

Different studies performed on melanoma and metastatic renal-cell carcinoma (mRCC) patients under anti-PD-1 treatment showed that neutrophil-to-lymphocyte ratio values ≥5 were are strongly associated to shorter survival, thus assessing its potential use as a strong prognostic, and maybe predictive, biomarker (54, 63–67). Other retrospective studies in cancer patients showed that high NLR values during or after Nivolumab treatment, but not at baseline, were significantly associated with a worse outcome (27, 55, 68–71). The predictive value of NLR was recently formally assessed in a cohort of mUC patients, in which associations between candidate biomarkers and clinical benefit were investigated comparing a cohort treated with anti-PD-1/anti-PD-L1 to a cohort treated with taxanes. A NLR <5 and a high single nucleotide variant count were proposed as independent predictors of treatment response for ICIs (72).

The ANC, if above a certain threshold, also results negatively associated with treatment response either at baseline (73, 74) or on-treatment for different malignancies (27, 60, 75). In this context, Pan et al. observed that metastatic melanoma patients with high baseline ANC were more likely to undergo disease progression than patients with low values (73). Additionally, Nivolumab decreased the levels of ANC in the responder group of another cohort of melanoma patients, mirroring the decrease in systemic inflammation levels (75). Multiple mechanisms by which neutrophils may boost cancer growth have been proposed, including the release of immunosuppressive cytokines and chemokines that affect the recruitment and phenotype of different immune cells. Neutrophils can also exert their immunosuppressive function through production of arginase 1 (ARG1) and ROS. Arginase depletes a fundamental nutrient, arginine, from the surrounding environment, leading to the inhibition of T cell proliferation and function, while ROS can suppress lymphocyte activation and, at high concentrations, induce T cell apoptosis (76).

Interestingly, within the granulocytic fraction, eosinophils seem to have a protective role and are usually associated with favorable treatment outcomes (54, 74, 77). Even if their predictive role remains unclear, a relative eosinophil count <1.5%, as well as elevated baseline levels of LDH and CRP, were independently associated with poor OS in a cohort of uveal melanoma patients undergoing anti-PD-1 monotherapy (78). A possible explanation behind the positive effect of eosinophils is their capacity to recruit cytotoxic T lymphocytes through CCL5, CXCL9, and CXCL10, to induce an anti-tumor phenotype in macrophages through TNFα and IFNγ and to normalize the tumor vasculature (79).

To conclude, several reports indicate that MDSCs, monocytes and neutrophils may reflect a compromised inflammatory status and thus be a reliable negative predictor of response to anti-PD-1 therapy. Nonetheless, some works underline how a high monocyte frequency could have a positive impact on patient outcome since it may mirror a macrophage-mediated cell cytotoxicity at tumor site. Moreover, the role of granulocytes is also debated, since eosinophils appear to have a protective role given their ability to recruit cytotoxic T lymphocytes.

### Tumor Biomarkers

#### Monocyte/Macrophage Lineage

One of the first papers comparing the tumor transcriptome at baseline of melanoma patients undergoing anti-PD-1 therapy described an “innate anti-PD-1 resistance signature” that comprised genes involved in the mesenchymal transition (*AXL, ROR2, WNT5A, LOXL2, TWIST2, TAGLN, FAP*), immunosuppression (*IL10, VEGFA, VEGFC*) and monocyte and macrophage chemotaxis (*CCL2, CCL7, CCL8*, and *CCL13*) in non-responder patients (80). This signature was associated with resistance to anti-PD-1, but not with anti-CTLA-4, in three additional melanoma cohorts and was also described in other tumor types, where its predictive value was however not proven. In another paper describing predictive gene signatures related to T cell dysfunction and T cell exclusion (TIDE) in melanoma patients, the presence of TAM and MDSC signatures, along with cancer-associated fibroblasts, was related to reduced T cell infiltration and resistance to anti-PD-1 and anti-CTLA-4 (29). Moreover, Neubert et al. observed that IFNγ and TNFα produced by antigen-specific CD8^+^ T cells induced the macrophage-colony stimulating factor CSF-1 in melanoma cells, possibly recruiting and activating TAMs. Circulating CSF-1 levels in melanoma patients are significantly higher than in healthy donors and positively correlated with disease progression. In the same cohort of melanoma patients treated with anti-PD-1 analyzed in (80), the authors observed a co-enrichment of CD8^+^ T cells with CSF-1 or various TAM-specific markers in pre-treatment biopsies of non-responders, suggesting that the recruitment of CD163^+^ M2-like macrophages by activated lymphocytes might be a mechanism of resistance to immunotherapy. Interestingly, in the presence of IFNγ and TNFα melanoma cells can also upregulate *TGFB, IL10, VEGFA*, and *VEGFC* genes, which can further modulate the immunosuppressive phenotype of TAMs (81). A recent retrospective analysis in NSCLC patients uncovered an epigenetic signature, called EPIMMUNE, predictive for PFS upon anti-PD-1 treatment. The authors observed that EPIMMUNE-negative tumors, prevalent among non-responders, were particularly infiltrated by macrophages and neutrophils, in contrast with EPIMMUNE-positive biopsies, characterized by a strong lymphoid infiltrate (82). CD73^hi^ myeloid cells overexpressing several chemokine receptors and immunosuppressive factors are also highly abundant in the TME of glioblastoma, where they are negatively correlated with OS. These cells persist in glioblastoma patients after Pembrolizumab and hamper the efficacy of anti-PD-1 and anti-CTLA-4 in murine models (83).

Another interesting report of a predictive negative role of macrophages in ICI therapy regards the phenomenon of hyperprogression, an accelerated growth of tumors observed in 9–29% of the patients under ICIs that is still poorly understood. In baseline biopsies of a small cohort of NSCLC cancer, Lo Russo et al. have described that a subtype of clustered CD163^+^CD33^+^PD-L1^+^ macrophages with epithelioid morphology was significantly enriched in all hyperprogressor patients compared to patients not experiencing hyperprogression (84).

Another important aspect that should be considered when analyzing the TME is the *localization* of the different cell types. On a small cohort of melanoma patients treated with anti-PD-1, non-responders displayed a significantly higher proximity of CD68^+^ myeloid cells to CD8^+^ T cells compared to responders in pre-treatment and on-treatment biopsies (85). Intriguingly, long-lasting contacts between macrophages and CD8^+^ T cells in surgically resected NSCLC tumors are associated with impaired motility and reduced infiltration of lymphocytes in tumor islets; in pre-clinical models resistant to anti-PD-1, the concomitant depletion of macrophages can restore T cell motility and infiltration into tumor islets with increased tumor cell killing, suggesting that myeloid cells can modulate the TME not only through soluble mediators but also by physical contact with the surrounding cells (86).

Given the plasticity of myeloid cells and the differences in the microenvironment among tumors, the phenotype of this lineage can greatly vary. The simple abundance of CD68^+^ cells, classically considered to represent macrophages, is thus rarely informative, while the *functional orientation* of myeloid cells by multiparametric IHC, flow cytometry or RNA sequencing allows to define a clearer relationship between the distinct subsets and the clinical outcome (32). Therefore, functionally different myeloid subpopulations can have an opposite effect on the response of patients to ICIs. In some reports, for instance, the presence of PD-L1^+^ macrophages seems more valuable as a predictive biomarker than the abundance of macrophages *per se*. Clinical responses are linked to high expression of PD-L1 in macrophages and dendritic cells in melanoma patients treated with a combination of anti-CTLA-4 and anti-PD-1 and in ovarian cancer patients treated with anti-PD-1, even if in the latter case the results are not statistically significant due to poor responses (87). Similarly, in the SARC028 trial, sarcoma patients who had an objective response to Pembrolizumab had a significantly higher average percentage of tumor cells and TAMs expressing PD-L1 at baseline compared to non-responders (88). Moreover, high counts of PD-L1^+^ macrophages, but not PD-L1^+^ tumor cells, were predictive of better OS after anti-PD-1 or anti-PD-L1 therapy in NSCLC (89).

In melanoma patients treated with anti-PD-1, anti-CTLA-4 or the combination, myeloid cells were enriched in non-responder lesions (90). However, when the myeloid compartment of the same cohort was analyzed in detail by single-cell RNA sequencing (scRNAseq), TAMs of responders were found to express CXCL10 and CXCL11 which, together with CXCL9, are predictive markers of response to anti-PD-1 and anti-PD-L1 in metastatic melanoma and mUC. The authors described distinct gene expression profiles in macrophages from responder and non-responder patients, unveiling other potential markers of response and resistance that could help understand the complex biology of the myeloid compartment in ICI therapy (91).

In another recent report, peripheral T cell and M1 macrophage signatures have shown to be enriched in NSCLC patients that displayed durable clinical benefit after anti-PD-1 treatment compared to non-responders. In particular, PFS was longer in patients with high peripheral T cell or M1 signatures, but OS was not significantly different. The same authors observed that these signatures behaved similarly in metastatic melanoma patients treated with Nivolumab [analyzed in (92)], although the differences between responders and non-responders did not reach statistical significance (93).

These examples demonstrate the importance of a more detailed analysis of the TME by multiparametric flow cytometry, multidimensional IHC, as well as scRNAseq, to better understand the role of each cell subset as biomarkers for immunotherapy.

Data regarding human myeloid cells during or after treatment are limited and conflicting. As an example, in two different studies with melanoma patients treated with Nivolumab or Pembrolizumab, responder biopsies displayed an increase in CD8^+^ T cells in both studies, but in one case there was a reduction in the macrophage transcriptomic signature (92), while in the other there was an increase in peritumoral CD68^+^ macrophages after treatment (48). However, in the latter cohort there was a significant increase in PD-L1 expression in macrophages of responder patients, suggesting that the function of these cells might be modified after anti-PD-1 therapy (48).

Neoadjuvant immunotherapy, currently explored for some tumor indications to ameliorate the efficacy of surgical resection, is giving important insights into the activity of ICIs and pharmacodynamic biomarkers. In a cohort of advanced melanoma patients that received neoadjuvant Pembrolizumab before resection, anti-PD-1 therapy provoked an increase in CD8 TILs and an upregulation of PD-L1 and other genes involved in T cell activation and migration. While these parameters were associated with clinical benefit, an increase in CD163^+^ myeloid cells and a decrease in CD3^+^ lymphocytes were observed in patients that recurred after surgery. In one of these relapsing patients, Nanostring analysis revealed the presence of T cell activation transcripts but also the presence of a myeloid signature (comprising *CD14, CCL8, CXCL14, CLEC5A*, and *CSF1R* genes), confirming the immunofluorescence data (94). Moreover, the same patient experienced p53 loss at recurrence, an event that could further increase immunosuppression, as p53 activation has been linked to MDSC reduction and anti-tumor immunity in mice (95). In another cohort, neoadjuvant Nivolumab in resectable NSCLC induced necrotic areas with large infiltrates of lymphocytes and foamy macrophages in tumors of patients with major pathological response at surgery (96, 97).

In glioblastoma patients treated with neoadjuvant anti-PD-1, increased T cell infiltration and chemokine transcripts have been described, even though there was no clear clinical benefit. In these patients no obvious modulations of myeloid cells have been observed (98), in line with what had been reported in glioblastoma patients after Pembrolizumab (83).

#### Granulocyte Lineage

In two small cohorts of metastatic melanoma treated with anti-PD-1, the infiltration of neutrophils, but not of macrophages, was higher in patients with progressive disease relative to those with clinical responses. The authors have shown that in murine models the infiltration of macrophages and neutrophils are, at least in part, mutually regulated and are also influenced by tumor-intrinsic factors, thus pointing at the need of a better understanding of the cross-talk between different cells subsets in the TME (99). However, the role of TANs or other tumor-infiltrating granulocytes in ICI treatment still needs to be clarified.

These results suggest that, at least in some tumor types, on-treatment myeloid cell density and phenotype in the TME might be potentially used as both predictive and pharmacodynamic biomarkers.

## Anti-PD-L1 Inhibitors

Three drugs are currently approved to target PD-L1: Durvalumab, Avelumab and Atezolizumab. These antibodies are approved for fewer indications than anti-PD-1 blockers, i.e., mUC, Merkel Cell carcinoma, NSCLC and, in combination with chemotherapy, triple-negative breast cancer and small cell lung cancer, with less reports so far about the predictive role of myeloid cells on the clinical response.

### Circulating Biomarkers

Bocanegra et al. analyzed the systemic differences in PD-L1 expression that could explain the opposite response of two patients with PD-L1-negative NSCLC tumors treated with Atezolizumab. PBMCs were divided into CD11b^high^ (monocytes, M-MDSCs and neutrophils), CD11b^low^ (DCs, PMN-MDSCs, some T cells and NK cells), and CD11b^−^ (T and B lymphocytes and plasmacytoid DCs) subsets. Responding patients exhibited high percentages of PD-L1^+^ cells in CD11b^−^ and CD11b^high^ immune cell types, but not in CD11b^low^ cells. To confirm and extend these data, other NSCLC patients under PD-1/PD-L1 blockade were analyzed, showing a significant association between a high percentage of circulating PD-L1^+^CD11b^+^ cells at baseline and an objective clinical response. Moreover, a tendency for responders to express high amount of PD-L1 within CD11b^+^CD14^+^ cells was observed, although it did not reach statistical significance. Intriguingly, patients with high percentages of circulating memory CD4^+^ T lymphocytes and low percentages of PD-L1^+^ immune cells did not respond to ICIs, highlighting the relevance of PD-L1 expression by myeloid cells in predicting treatment efficacy (100).

In advanced NSCLC patients treated with Atezolizumab, disease control was associated with decreased frequencies of Tregs and Lin^−^HLA-DR^−^CD33^+^CD11b^+^ MDSCs and a reduction in NLR after treatment (101).

In mUC patients, successive doses of Atezolizumab and Avelumab correlated with a significant decrease of PD-L1^+^ M-MDSC and PD-L1^+^ eMDSC (CD33^+^HLA-DR^low/−^CD14^−^CD15^−^) after the first dose. However, changes in PD-L1 expression in MDSCs either before or after therapy did not predict and neither correlate with ICI response, showing the need for further studies to find predictive biomarkers in mUC patients (52).

In a clinical trial involving patients with metastatic prostate cancer treated with the PARP inhibitor Olaparib combined with Durvalumab, a baseline Lin^−^HLA-DR^−^CD11b^+^CD33^+^ MDSC fraction lower or equal to the median of the group correlated with longer PFS. In this study, myeloid cells were also useful on-treatment markers, as patients with increased expression of CD83 on CD141^+^ mDC after treatment had prolonged PFS (102).

A case study report of a NSCLC patient treated with Durvalumab as a maintenance therapy after chemotherapy and radiotherapy showed a 3-fold drop in the level of IL-4Rα^+^ M-MDSC and in the expression of the *CD274* (PD-L1), *PTGS2, IL10*, and *IDO1* genes in PBMCs after two administrations of the anti-PD-L1 antibody, accompanied by a reduction in the suppressive potential of these cells compared to baseline. After 6 months of Durvalumab, this patient is still in clinical and radiologic disease remission (103).

As previously discussed, it should be considered that none of these studies compared anti-PD-L1 therapy to other treatments. Further investigation is thus needed to clarify the predictive and/or prognostic role of myeloid cells in this context.

### Tumor Biomarkers

Compared to anti-PD-1, the amount of information regarding tumor biomarkers under anti-PD-L1 antibodies is still limited. In a clinical trial with mRCC patients, a myeloid signature comprising *IL6, CXCL1, CXCL2, CXCL3, CXCL8*, and *PTGS2* genes has recently been proposed as a resistance mechanism to Atezolizumab. Atezolizumab alone was more effective in patients with tumors enriched in cytotoxic T cells (T_eff_^high^) that were also Myeloid^low^. In the T_eff_^high^ Myeloid^high^ subgroup, the combination of Atezolizumab with the anti-VEGF Bevacizumab showed better activity than Atezolizumab alone, suggesting that the inhibition of VEGF could counteract the presence of immunosuppressive myeloid cells (104).

In addition, myeloid-associated genes (*COX2, IL8, IL1B*) in the tumor and circulating cytokines (IL-8 and IL-6) were associated with resistance and shorter OS in urothelial bladder cancer patients treated with anti-PD-L1 (Atezolizumab or Durvalumab) (105–108).

In summary, myeloid cells have been investigated as predictive markers of response to anti-PD-L1 treatment only in few studies, comprising different tumor types and a variety of myeloid subsets, hindering up to now the definition of biomarkers clearly correlated to patient response.

## Anti-CTLA-4 Inhibitors

CTLA-4 is another immune checkpoint that mediates the physiological inhibition of activated T cells by competing with CD28 for the binding of CD80 and CD86 costimulatory molecules on antigen-presenting cells (APCs). Two monoclonal anti-CTLA-4 antibodies, Ipilimumab and Tremelimumab, are currently used in cancer to release the brake induced by CTLA-4 and build an effective immune response. Ipilimumab has been approved by the FDA for metastatic melanoma and, in combination with Nivolumab, for RCC and MSI CRC. Tremelimumab has been evaluated in the treatment of melanoma, mesothelioma, NSCLC, head and neck squamous cell carcinoma, prostate, pancreatic and hepatocellular carcinomas. Initial phase I and II studies of Tremelimumab in metastatic melanoma were promising, but a phase III trial was stopped because the antibody did not demonstrate superiority to standard chemotherapy, although responses were more durable (109).

### Circulating Biomarkers

#### Monocyte Lineage

Many papers report the role of MDSCs as predictive markers for anti-CTLA-4 treatment, especially in Ipilimumab therapy. Among the three MDSC subsets, CD14^+^HLA-DR^low/−^ M-MDSCs are more commonly associated with resistance to this treatment. In regionally advanced melanoma patients treated with neoadjuvant Ipilimumab, circulating CD14^+^HLA-DR^low/−^ M-MDSC levels were lower at baseline but tended to increase, although not reaching statistical significance, in the relapse-free group, while frequencies in the relapsed group remained stable (110). Another work on malignant melanoma patients treated with Ipilimumab showed similar results: patients having distant metastasis in the skin or lymph nodes had lower levels of Lin^−^CD14^+^HLA-DR^−^ M-MDSCs compared to patients having distant metastasis in vital organs or increased LDH. When comparing responders to Ipilimumab with non-responders, significantly lower percentages of Lin^−^CD14^+^HLA-DR^low/−^ M-MDSCs were observed in the former group (111). Similarly, a high baseline frequency of M-MDSCs and high levels of IL-6 were associated with a reduced response to Ipilimumab in melanoma patients (112).

A study partially contradicting these results showed no significant differences between baseline levels of M-MDSCs in patients with clinical benefit and those with progressive disease. However, after 3 and 9 weeks from Ipilimumab administration, patients with clinical benefit showed lower frequencies of this cell population, while no significant changes were observed in patients with progressive disease. Moreover, at week three after Ipilimumab M-MDSC were inversely correlated to survival (113). A low frequency of M-MDSCs was associated with long-term survival in another study on metastatic melanoma patients treated with Ipilimumab. The 2-years survival probability after Ipilimumab was 34.5% for patients with low MDSC frequency, while there were no survivors among patients with higher baseline levels.

A low AMC and a low frequency of CD14^+^ monocytes were also strongly associated with a favorable outcome. A combination model was defined including LDH, MDSCs, Relative Lymphocyte Count, AMC, and Absolute Eosinophil Count, where each of them also remained in the model as a significantly independent biomarker (114).

In melanoma patients treated with Ipilimumab, the baseline number of monocytes and neutrophils was significantly higher in non-responder patients compared to responders. Interestingly, before treatment, non-responders displayed a tendency for an increased frequency of CD14^+^CD11b^+^HLA-DR^low/−^SSC^low^ M-MDSCs as compared to responders and this difference became significantly higher upon the first Ipilimumab infusion. Moreover, M-MDSCs in responders were strongly reduced after the first infusion, whereas they increased upon the second Ipilimumab cycle in non-responders. CD15^+^CD11b^+^HLA-DR^low/−^SSC^low^ PMN-MDSC levels were also evaluated, but no differences were detected between the two groups of patients. Interestingly, the level of intracellular nitric oxide was significantly elevated in M-MDSCs from non-responders compared to responders and higher M-MDSC percentages in non-responders significantly correlated with elevated nitric oxide production in these cells upon the first Ipilimumab infusion. Moreover, PD-L1 expression was downregulated in PMN-MDSCs of responders after the first Ipilimumab dose. Besides MDSCs, a significant increase in the eosinophil count after treatment was associated with an improved clinical response (115).

An interesting work has also demonstrated that miRs inducing MDSCs could represent predictive markers of response to ICIs in advanced melanoma patients (5). In this study, extracellular vesicles potentially derived from melanoma tumor cells were able to convert healthy donor monocytes into MDSCs (EV-MDSCs), by downregulating HLA-DR at the RNA level. Monocytes skewing to EV-MDSCs also showed changes in miR expression as compared to normal monocytes. MiR-146a, miR-146b, let-7e, miR-99b, and miR-125b were enriched in the extracellular vesicle fraction responsible for MDSC generation and were found to modulate the phenotype and function of monocytes toward MDSCs *in vitro*. In metastases from melanoma patients, high levels of miR-146a, miR-155, miR-125b, miR-100, let7e, miR-125a, miR-146b, and miR-99b were detected and correlated with CD163, CD14, CD209, CD68, ITGAM, and CD33 myeloid markers. An increased level of MDSC-miRs was detected in plasma samples from melanoma patients with advanced disease. A retrospective analysis in metastatic melanoma patients treated either with Ipilimumab, Nivolumab, or targeted therapy showed that patients with a low miR-score had a significantly longer OS, thus underlying the prognostic (and maybe predictive) value of these M-MDSC-inducing miRs (116).

Given the relevance of M-MDSCs as predictive biomarkers for response to Ipilimumab, Kitano et al. proposed a computational algorithm-driven analysis of PBMCs, demonstrating that melanoma patients with a pre-treatment M-MDSC frequency lower than 14.9% had a significantly longer OS and that M-MDSC levels inversely correlated with peripheral CD8^+^ T cell expansion following Ipilimumab treatment (56). Beside melanoma patients, this algorithm will constitute a useful tool to evaluate M-MDSC frequencies in other tumor types.

High pre-treatment levels of M-MDSCs were also associated with reduced OS in castration-resistant prostate cancer treated with combined GVAX/Ipilimumab immunotherapy. In these patients, treatment-induced activation of conventional cDC1 and cDC3 dendritic cells was associated with prolonged OS, but also an increased risk of immune-related adverse events. In an unsupervised cluster analysis, patients with low pretreatment M-MDSCs, high pretreatment CD4^+^CTLA-4^+^ T cells and high levels of cDC1/cDC3/monocyte activation during treatment displayed prolonged survival (117).

CD14^+^IL-4Rα^+^ M-MDSCs were identified by Damuzzo et al. as negative predictors of response to Ipilimumab. In this study, four MDSC subsets were analyzed in the PBMCs of advanced melanoma patients, at baseline and 12 weeks after Ipilimumab: CD14^+^IL-4Rα^+^ M-MDSCs, CD14^+^HLA-DR^low/−^ M-MDSCs, Lin^−^HLA-DR^−^CD33^+^CD11b^+^ eMDSCs, and CD15^+^IL4-Rα^+^ PMN-MDSCs. A significant expansion of the two subsets of M-MDSCs and of PMN-MDSCs was observed at baseline compared to healthy controls and, upon treatment, high levels of CD14^+^IL-4Rα^+^M-MDSCs were independent prognostic factors of reduced OS. Moreover, longer OS was associated with low levels of IL-6, CRP, S100B, and LDH at baseline and after treatment. In a multivariate survival model, high levels of LDH and CD14^+^IL-4Rα^+^M-MDSCs post-treatment were identified as negative independent markers of reduced OS, thus showing that the IL-4Rα^+^M-MDSC subset should be considered, together with CD14^+^HLA-DR^low/−^ cells, to select patients that could get most benefit from anti-CTLA-4 (118).

Interestingly, the population of non-classical monocytes has on the contrary been associated to a positive response to ICIs. In fact, advanced melanoma patients responding to Ipilimumab displayed the highest percentages and absolute counts of circulating non-classical monocytes at baseline. The authors showed that non-classical CD14^+^CD16^+^monocytes, but not classical CD16^−^ monocytes, were able to lyse Tregs *ex-vivo* through CD16-Fc-mediated antibody-dependent cellular cytotoxicity (ADCC) mediated by Ipilimumab (119).

In metastatic melanoma patients, low levels of CD33^+^CD11b^+^HLA-DR^−^ MDSCs before Ipilimumab correlated with an objective clinical response, long-term survival, increased CD3ζ chain expression in T cells and an improved clinical status. Conversely, patients with more than 55.5% circulating MDSCs had a significantly shorter OS (120). In a neoadjuvant study with Ipilimumab in advanced melanoma, the treatment induced an expansion of activated CD4^+^ and CD8^+^ melanoma-specific T cell clones, an increase in circulating Treg, with greater Treg increase associated to improved PFS, and a reduction in all MDSC subsets, especially the M-MDSC fraction. A greater decrease in the circulating HLA-DR^−^CD33^+^ MDSC population was related to improved PFS in this cohort (121).

#### Granulocyte Lineage

Concerning PMN-MDSCs, this cell population is less frequently associated with response to Ipilimumab. Only the level of PD-L1 in PMN-MDSCs has been reported to be lower in responders as compared to non-responders in (115). In a cohort of melanoma patients treated with Ipilimumab, the level of PMN-MDSCs decreased after the first dose of Ipilimumab, but no information is given about the impact on treatment response (122). Another work on advanced melanoma patients treated with Ipilimumab showed that patients with high ANC and dNLR at baseline had an increased risk of death or disease progression (123).

Besides MDSCs, the relative eosinophil count, together with an elevated serum LDH and CRP, was significantly associated with survival in metastatic uveal melanoma patients treated with combined Ipilimumab and anti-PD-1 (78).

In conclusion, the collected data point mostly to the monocytic subsets, particularly CD14^+^HLA-DR^+^ M-MDSCs, CD14^+^IL4-Rα^+^M-MDSCs and non-classical monocytes, as useful markers for the selection of patients that could benefit more from Ipilimumab immunotherapy.

### Tumor Biomarkers

For anti-CTLA-4 Ipilimumab, the predictive value of tumor biomarkers remains to be consolidated. The TIDE and TIS transcriptomic signatures, as well as genes linked to T cells cytotoxicity, Th1 chemokines and antigen presentation seem useful for the identification of responders among melanoma patients (29, 124). The mutational and neoantigen load (125) and a high ratio of CD8^+^ density in the intratumoral region have also been related to clinical benefit to Ipilimumab in melanoma (126), while PD-L1 staining by IHC alone does not seem to be predictive (127).

#### Monocyte/Macrophage Lineage

Even if few studies exist on the predictive role of myeloid subsets, Capone et al. observed that BRAF WT melanoma patients with durable clinical benefit from Ipilimumab had a reduced transcriptomic “myeloid score.” In the same cohort, the downregulation of CD73 gene, expressed by tumor-infiltrating myeloid cells as previously discussed, was also associated with response, irrespective of the BRAF status (128).

Interestingly, macrophage infiltration at baseline was even more useful than CD8 density in the distinction of responders vs. non-responders in a small cohort of melanoma patients treated with Ipilimumab. Responders displayed a higher CD68^+^/CD163^+^ ratio and higher CD68^+^CD16^+^ density at baseline than non-responders, with a concomitant reduced infiltration of CD163^+^CD16^+^ macrophages. The authors hypothesized that the enrichment of “inflammatory” CD68^+^CD16^+^ macrophages over immunosuppressive CD163^+^ macrophages could create a more favorable TME for the anti-tumor activity of Ipilimumab. Moreover, post-treatment tumor biopsies from responders had lower Treg infiltration than lesions from non-responder patients and the authors hypothesized that this could be explained by the increased presence of FcγR “inflammatory” macrophages capable of inducing ADCC in the presence of the IgG1 Ipilimumab antibody and depleting Tregs (119). This hypothesis is also supported by Arce Vargas et al., who demonstrated how human Fcγ receptors expressed by myeloid cells can induce ADCC after binding to a chimeric murine anti-CTLA-4 with a human IgG1 Fc, *in vitro* and in human-FcγR murine models. In addition, melanoma patients with high TMB and the CD16-V158F polymorphism (conferring higher binding affinity to IgG1 antibodies) had higher response rates than all the other patients, suggesting that FcγR^+^ myeloid cells in the tumor might contribute to the anti-tumor activity of Ipilimumab through ADCC-dependent Treg depletion (129).

The localization of different subpopulations in the TME (tumor region, stroma, invasive margin) is crucial for response, in addition to their phenotype and functional status. Madonna et al. observed that melanoma patients having baseline biopsies with few CD8^+^ T cells combined with high numbers of CD163^+^PD-L1^+^ macrophages at the invasive margin survived significantly longer than any other group upon Ipilimumab (127).

Little information is currently available for on-treatment tumor biomarkers for anti-CTLA-4 therapy. A study of neoadjuvant Ipilimumab in advanced melanoma showed an increased infiltration of memory CD4^+^ and CD8^+^ T cells in the TME. At the same time, a reduction in tumor-infiltrating Tregs was associated with response or stable disease and decreased levels of tumor HLA-DR^−^CD33^+^CD11b^+^ MDSCs after treatment were associated with a longer PFS. As mentioned in the previous section, in these patients an association between reduction in systemic HLA-DR^−^CD33^+^CD11b^+^ MDSC and improved PFS was also observed (121).

As previously stated, the predictive vs. prognostic value of these biomarkers should be confirmed through randomized trials since the treatment with anti-CTLA-4 has not been formally compared to other treatments in most studies.

## Binding of ICIs to Myeloid Cells: Potential Mechanisms of Action and impact on Biomarkers

Together with their well-known regulatory functions on lymphocytes extensively reviewed elsewhere (6, 7, 22, 23, 35), circulating and tumor-infiltrating myeloid cells can express PD-L1, PD-1 and Fcγ receptors (FcγR) and can thus directly bind to ICIs and modulate their activity. On the other side, ICIs can affect the phenotype of myeloid cells either through a direct binding to these cells or through the indirect effects of IFNγ and other mediators released by activated lymphocytes. Even though murine and human myeloid subsets are identified through different markers, preclinical models are crucial for the understanding of the mechanistic role of these cells in immunotherapy.

As for other antibody-based therapies, an important aspect to consider for ICIs is the expression of CD16, CD32, and CD64 FcγRs on the surface of myeloid cells. Depending on the isotype, the backbone of the mAb and the FcγR, the binding of the Fc part of antibodies can have different consequences. As reported before, the binding of Ipilimumab (119, 129), a potentially depleting IgG1, to the FcγR of myeloid cells can lead to the elimination of CTLA-4^+^ Tregs through ADCC or antibody-dependent phagocytosis (ADCP). In this context, the infiltration of the TME by myeloid cells can potentially be a positive predictive biomarker for Ipilimumab therapy.

On the contrary, most anti-PD-1 and anti-PD-L1 antibodies have low or significantly reduced binding to FcγR to avoid potential ADCC and complement-dependent cytotoxicity (CDC), especially when the target molecule is expressed on effector T cells. In murine tumor models, the anti-PD-1 antibody can be transferred from CD8^+^PD-1^+^ T cells to PD-1^−^ macrophages through FcγRIIb/III receptors and the same phenomenon can be reproduced *in vitro* with human cells and Nivolumab. Besides, the use of FcγRIIb/III blocking antibodies prior to anti-PD-1 improved its anti-tumor efficacy in mice (130). In a similar way, another paper showed that a human IgG4 anti-PD-1 antibody, bearing an S228P mutation as most approved immune checkpoint blockers, mediated a crosslink with FcgRI(CD64)^+^ macrophages, resulting in the activation, rather than inhibition, of PD-1 signaling in T cells, the elimination of PD-1^+^ CD8 cells by ADCP and increased secretion of IL-10 by macrophages. Compared to an identical anti-PD-1 antibody that lacked FcγR binding, the S228P-mutated antibody displayed a reduced anti-tumor effect *in vivo*, highlighting the potential role of FcγR-expressing myeloid cells in the negative regulation of ICIs (131). These observations need to be considered for the drug development of antibodies because, even though most mAbs are IgG4 or mutated IgG1 with no or low ADCC, ADCP, or CDC, they can still bind to different FcγR with unclear clinical consequences. Further studies are needed to elucidate whether the described mechanisms can also be observed in patients, further supporting the predictive role of specific subsets of myeloid cells in anti-PD(L)1 therapy.

In addition to this, several preclinical experiments have tried to shed light on the impact of ICIs on myeloid cells, which can express PD-1 and PD-L1. ScRNAseq of MC38 tumors in immunocompetent mice treated with anti-PD-1 revealed an expected increase in IFNγ, immune checkpoints and costimulatory molecules in CD8 lymphocytes in responders. Interestingly, this was accompanied by an enrichment in an M1-like signature including *HLA-DR, CXCL9, CXCL10, CCL5, CCL8*, and *STAT1* transcripts, while, conversely, an M2-like signature comprising *SPP1, PTGS1, MRC1, MSR1, ARG1*, and *CCR2* mRNA was observed in non-responders (132). In the T3 murine model, progressing tumors are highly enriched in CD206^+^ macrophages, but anti-PD-1, anti-CTLA-4 or combination treatment induced the accumulation of iNOS^+^ inflammatory macrophages. ScRNAseq and mass cytometry further confirmed the transformation of control tumors, mainly infiltrated by CCR2^+^ monocytes and CX3CR1^+^CD206^+^CCL2^+^CD49d^+^ macrophages, into tumors enriched in iNOS^+^PD-L1^+^CXCL2^+^ cells after ICI treatment (133). In a similar way, Dhupkar et al. have shown that anti-PD-1 treatment induced a significant reduction in lung metastases and a decrease in PD-L1 expression by metastatic tumor cells in human LM7 osteosarcoma-bearing mice. In this T cell-deficient model, NK and macrophages were PD-1^+^ and their fraction was increased in the tumor after treatment; the anti-tumor effect of anti-PD-1 blockade was lost after macrophage, but not NK cell, depletion. Moreover, anti-PD-1 provoked an increase in CD86 and a reduction in CD163 staining in lung metastases compared to control mice, suggesting a shift from M1-like to M2-like macrophages (134).

PD-1^+^ macrophages have also been described in mice and human colorectal cancer (CRC), where they display M2-like features (CD206^+^CD64^+^ large, foamy macrophages with uncleared phagocytic material) and are involved in tumor growth and invasion. In the CT26 model these macrophages had reduced phagocytosis compared to the PD-1^−^ counterpart, restored by the knock-out of PD-L1 on tumor cells. In immunodeficient mice bearing a PD-L1 human CRC xenograft, anti-PD-(L)1 inhibitors were able to reduce tumor growth and this effect was abrogated by macrophage depletion (135). PD-1^+^ macrophages with an M2-like phenotype have also been described in NSCLC biopsies and in the murine LLC model. In murine tumors, PD-1^+^ macrophages have a distinct transcriptomic profile compared with PD-1^−^ cells. In NSCLC tumors, PD-1^+^ macrophages are mainly stromal CD163^+^ macrophages and are associated with poor prognosis, suggesting once more that the phenotype and the localization of myeloid cells are likely crucial parameters to take into account for ICI biomarkers (136).

In a similar way, Hartley et al. investigated the direct effect of anti-PD-L1 antibodies on PD-L1^+^ macrophages at the tumor site, given the prevalence of this cell subset in human tumors. The authors discovered that the treatment of murine and human macrophages with anti-PD-L1 antibodies increased their proliferation, survival and activation, as measured by the upregulation of CD86, MHC II, CD40, TNFα, and IL-12 and of several transcripts linked to myeloid inflammation. The same effects could be induced when macrophages were pre-treated with anti-FcRII/III antibodies. The authors have hypothesized that PD-L1 provides a constitutive negative signal in macrophages that can be reversed by anti-PD-L1 antibodies. The anti-PD-L1 treatment in syngeneic mouse models increased the number of TAMs and upregulated CD86 and MHC II, even in the absence of T cells. The authors also showed that the *in vivo* combination of anti-PD-1 and anti-PD-L1 antibodies, given their non-redundant effects, is more effective than either monotherapies in mice (137).

These preclinical experiments suggest that PD-1 and PD-L1 could negatively signal in macrophages, keeping them in a non-inflammatory, non-phagocytic state. If confirmed in the clinical setting, this could imply that anti-PD-(L)1 immunotherapy, together with its effect on T cells, might also cause an enrichment of M1-like macrophages either directly (though the binding to PD-1 or PD-L1) or indirectly (through cytokines release by activated lymphocytes). This phenotypic and functional switch could also be used as a tumor pharmacodynamic biomarker for these antibodies.

## Combination Strategies for Improving ICI Therapy by Targeting Myeloid Cells: an Overview of Clinical Data

Despite the great success of ICIs, the large majority of patients present a *primary* (never-responders) or *acquired* resistance after a period of response (138), but to date the reasons remain largely unclear. Combinatorial approaches with drugs that target immunosuppressive networks have become attractive to extend the benefits of immunotherapy to non-responding patients and are currently being tested in various clinical trials, as shown in Table 1. Through the modulation of distinct cell subsets, these combinations can be useful to overcome primary, as well as acquired, resistance to ICIs.

**Table 1:** Clinical Trials of combinations of ICIs with myeloid-targeting drugs.

	**ICI**	**Drug**	**Target**	**Phase**	**Clinical trial**	**References**
α-PD-1	Nivolumab Pembrolizumab	Cabiralizumab Cabiralizumab Pexidartinib ARRY-382 AMG 820	CSF1R	Phase 1 Phase 2 Phase 1/2 Phase 1/2 Phase 1/2	NCT02526017 NCT03336216 NCT02452424 NCT02880371 NCT02713529	(139) (140) (141) (142) -
α-PD-L1	Atezolizumab Durvalumab	Emactuzumab Pexidartinib	CSF1R	Phase 1 Phase 1	NCT02323191 NCT02777710	- (143)
α-PD-1	Pembrolizumab	LY3475070	CD73	Phase 1	NCT04148937	-
α-PD-L1	Atezolizumab Durvalumab	TJ004309 MEDI9447	CD73	Phase 1 Phase 1	NCT03835949 NCT02503774	- (144)
α-PD-1	Spartalizumab	PBF-509	Adenosine-A2A Receptor	Phase 1/2	NCT02403193	(145)
α-PD-L1	Atezolizumab Durvalumab	Ciforadenant (CPI-444) AZD4635	Adenosine-A2A Receptor	Phase 1 Phase 1	NCT02655822 NCT02740985	(146, 147) (148)
α-PD-1–α-CTLA-4	Nivolumab-Ipilimumab	VX15/2503 (Pepinemab)	Semaphorin 4D	Phase 1 Phase 1 Phase 1 Phase 1	NCT03690986 NCT03373188 NCT03425461 NCT03769155	(149) (149) - (150)
α-PD-L1	Avelumab	VX15/2503 (Pepinemab)	Semaphorin 4D	Phase 1/2	NCT03268057	(151)
α-PD-1	Nivolumab Pembrolizumab	Epacadostat Epacadostat	IDO-1	Phase 1 Phase 1/2 Phase 3 Phase 3 Phase 3 Phase 3 Phase 2 Phase 2 Phase 3	NCT03707457 NCT02178722 NCT02752074 NCT03260894 NCT03374488 NCT03358472 NCT03322540 NCT03322566 NCT03361865	- (152) (153) - (154) (155) (156) (157) (158)
α-PD-L1	Durvalumab	Epacadostat	IDO-1	Phase 1/2	NCT02318277	(159)
α-CTLA-4	Ipilimumab	Epacadostat	IDO-1	Phase 1/2	NCT01604889	(160)
α-PD-1	Nivolumab	IPI-549	PI3K-γ	Phase 1 Phase 2	NCT02637531 NCT03980041	(161)
α-PD-L1	Atezolizumab	IPI-549	PI3K-γ	Phase 2	NCT03961698	-
α-PD-1	Nivolumab	APX005M	CD40	Phase 1/2 Phase 1/2	NCT03214250 NCT03123783	(162) -
α-PD-1	Pembrolizumab Spartalizumab	MIW815 MK-1454 GSK3745417 MIW815	STING	Phase 2 Phase 1 Phase 1 Phase 1	NCT03937141 NCT03010176 NCT03843359 NCT03172936	- (163) - (164)
α-CTLA-4	Ipilimumab	MIW815	STING	Phase 1	NCT02675439	-
α-PD-1	Pembrolizumab	ATRA	Retinoic Acid Receptor	Phase 1/2	NCT03200847	-
α-CTLA-4	Ipilimumab	ATRA	Retinoic Acid Receptor	Phase 2	NCT02403778	(165)
α-PD-1	Nivolumab	Trabectedin		Phase 2 Phase 2	NCT03590210 NCT03886311	- (166)
α-PD-L1	Avelumab Durvalumab	Trabectedin Trabectedin		Phase 1/2 Phase 1	NCT03074318 NCT03085225	- -
α-PD-1–α-CTLA-4	Nivolumab-Ipilimumab	Trabectedin		Phase 1/2	NCT03138161	(167)
α-PD-1	Pembrolizumab	Axitinib	VEGF-R	Phase 1 Phase 3	NCT02133742 NCT02853331	(168) (169)
α-PD-L1	Avelumab	Axitinib	VEGF-R	Phase 3	NCT02684006	(170)
α-PD-1	Nivolumab	Bevacizumab	VEGF	Phase 1 Phase 2 Phase 2	NCT03382886 NCT03890952 NCT03452579	- - (171)
α-PD-L1	Atezolizumab	Bevacizumab	VEGF	Phase 1 Phase 1 Phase 2 Phase 3 Phase 3	NCT01633970 NCT02715531 NCT01984242 NCT02366143 NCT02420821	(172) (173) (104) (174, 175) (176)
α-CTLA-4	Ipilimumab	Bevacizumab	VEGF	Phase 1	NCT00790010	(177–181)
α-PD-1	Pembrolizumab	Trebananib	Angiopoietin-2	Phase 1	NCT03239145	-

Given the impact of myeloid cells on immunotherapy reported in the previous paragraphs, it seems reasonable to combine ICIs with drugs that target these subsets (3, 5, 7). A huge amount of preclinical data supports this hypothesis and the relevance of these combinations is also emerging in the clinic. In the following paragraphs, we discuss the main myeloid-targeting strategies designed to enhance the antitumor activity of ICIs by either decreasing the suppressive potential of myeloid cells, through the inhibition of their recruitment, differentiation or function, or boosting the anti-tumoral capabilities of specific myeloid subsets.

### Inhibitors of Colony-Stimulating Factor 1 Receptor (CSF-1Ri)

As previously discussed, TAMs and MDSCs are critical players within the immunosuppressive microenvironment. CSF-1 binds to the CSF-1R tyrosine kinase receptor on myeloid cells leading to myeloid cell proliferation, differentiation and recruitment into tumors (182). CSF-1/CSF-1R blockade promotes antitumor T cell responses and reduces tumor growth in several preclinical models in combination with immunotherapy, despite showing minor effects on tumor growth as a monotherapy (81, 86, 183, 184). Inspired by these encouraging preclinical results and by the first clinical results from CSF-1Ri monotherapy (185), several clinical trials combining ICIs with small molecules (as ARRY-382 or Pexidartinib) or mAbs (e.g., Emactuzumab or Cabiralizumab) directed against CSF-1R are currently ongoing in patients with solid tumors (Table 1).

Initial results from the combination of the anti-CSF-1R Cabiralizumab with Nivolumab showed a durable clinical benefit in heavily pre-treated patients with microsatellite stable pancreatic cancer. A durable depletion of circulating non-classical monocytes, a pharmacodynamic marker of Cabiralizumab and other CSF-1R targeting agents (186, 187), was observed with the Cabiralizumab monotherapy and the combination with Nivolumab (139), with a dose-dependent increase in the systemic levels of CSF-1 and IL-34 (CSF-1R ligands). Within tumors, a decrease from baseline of M2-like CSF-1R^+^CD163^+^ and total CD68^+^ macrophages, together with a concomitant increase in CD8^+^ effector T cells, was shown in patients treated with the combination. Furthermore, a significant increase in the expression of CSF-1R ligands and pro-inflammatory genes, associated with an M1-type polarization, were observed only in the tumors of responders to the combination (140). These results supported a Phase 2 study of a triple combination of Cabiralizumab plus Nivolumab with or without chemotherapy in advanced pancreatic adenocarcinoma (188). The results of ICI combinations with other CSF-1Ri are awaited to support the relevance of this promising approach.

### Inhibitors of CD73 and Adenosine Pathway

Apart from molecules that interfere with the myeloid cell recruitment, another interesting therapeutic approach is to target their ability to create an immunosuppressive environment. As previously described, CD73 is a myeloid marker that is emerging as an important modulator of the response to ICIs (83, 128). CD73 hydrolyses the adenosine monophosphate (AMP) into adenosine and inorganic phosphate. The increased expression of CD73 in TME directly associates with adenosine accumulation and exerts multiple immunosuppressive actions on the anti-tumor immunity (189, 190).

Adenosine signals through cyclic AMP that inhibits T cell receptor activation (191). Preliminary data shows that patient exposure to anti-PD-1/PD-L1 therapy increased the expression of adenosine A2A receptor (A2AR) and CD73, suggesting that the adenosine pathway might be a potential mechanism of resistance to ICIs (192). As previously mentioned, CD73 inhibition may be a useful strategy to improve the clinical outcome of glioblastoma patients treated with immunotherapies (83). As a matter of fact, the human anti-CD73 mAb MEDI9447 is currently being tested in a Phase I study as monotherapy and in combination with Durvalumab (144).

In a Phase 1/1b clinical trial, an oral small molecule inhibitor of A2AR (CPI-444) has shown anti-tumor activity in monotherapy and in combination with Atezolizumab in anti-PD-1/PD-L1 resistant and PDL-1-negative patients (146). CPI-444 induced CD8^+^ T cell infiltration into tumors and IFNγ- and Th1 signatures (192). The use of adenosine analogs or agonists on PBMCs has allowed to identify a transcriptomic “adenosine signature,” dominated by myeloid cytokines and chemokines, nearly identical to the “myeloid signature” associated with poor response to Atezolizumab in RCC patients (104). CPI-444 blocks the induction of these genes *in vitro* and seems to have a better anti-tumor activity in RCC patients with a high adenosine signature compared to patients with low expression (147).

### Anti-semaphorin 4D Antibodies

Semaphorin 4D (SEMA4D) is a transmembrane glycoprotein that binds to Plexin receptors, regulating the movement and differentiation of cells and displaying immunomodulatory effects in the TME (193). High levels of SEMA4D positively correlate with the presence of immunosuppressive TAMs and MDSCs, with concomitant exclusion of activated APCs and CD8^+^ T lymphocytes from the tumor (194). In preclinical models, blockade of SEMA4D was associated with an increased penetration of inflammatory F4/80^+^CD11c^+^ APCs and a decreased density of pro-tumorigenic CD206^+^ M2-like TAMs in the TME. Combination with anti–CTLA-4 led to tumor regression accompanied by enhanced T cell activity, increase in activated CD86^+^ monocytes in the tumor, augmentation of pro-inflammatory IFNγ, TNFα, and IL-6 and decrease in immunosuppressive MCP-1 and IL-10 cytokines (195). Based on these preclinical results, Pepinemab, a humanized anti-SEMA4D mAb, is currently being evaluated in combination with Ipilimumab and Nivolumab in solid tumors (149–151), as reported in Table 1.

### Inhibitors of Indoleamine 2,3-Dioxygenase 1 (IDO1)

Another important molecule involved in T cell immunosuppression in the TME is IDO1, which catalyzes the cleavage of L-tryptophan into kynurenine, leading to the inhibition of effector T cell proliferation and to the increase of Tregs (196, 197). IDO1 can be constitutively expressed by tumor cells or by macrophages, MDSCs and DCs in the tumor or the lymph nodes (198, 199) but can also be induced by inflammatory cytokines, such as IFN-γ, potentially inducing resistance to immunotherapy (200). High baseline IDO1 expression in tumors has been shown to predict response to anti-CTLA-4 in metastatic melanoma patients (201). In the B16 murine model, IDO1 inhibition combined with anti-CTLA-4 blockade resulted in increased infiltration of effector T cells, while attenuating Treg and MDSC accumulation (202). Expression of IDO1, PD-L1 and CTLA-4 in PBMCs of melanoma patients have been shown to be associated with a negative outcome, independently from disease stage (203). Based on these evidences, IDO1 inhibitors have been investigated for their potential to enhance the efficacy of ICIs.

Epacadostat is a highly selective oral inhibitor of IDO1 that induces enhanced proliferation of effector T cells and NK cells, increased activation of CD86^high^ dendritic cells and a contraction of human Tregs *in vitro* and murine Tregs *in vivo* (204, 205). Based on the encouraging results obtained in a Phase 1/2 study (152), several Phase 2 and Phase 3 trials (Table 1) were started to define the efficacy of the combination of Epacadostat with Pembrolizumab. However, in patients with advanced melanoma, the results of the Phase 3 study ECHO-301 were disappointing, with no improvement in PFS or OS in the combination vs. Pembrolizumab alone (153). Moreover, this study lacked biomarkers, which could have answered several key questions. In a recent review several explanations have been proposed for the negative outcome of ECHO-301, including a possible insufficient inhibition of IDO1, due to the inhibitor itself or the clinical dose, and the inadequate selection of patients; the authors however suggest to pursue the clinical development of inhibitors of IDO1, which still remains an attractive target for cancer immunotherapy (206).

### Inhibitors of Phosphoinositide 3-Kinase γ (PI3Kγ)

The PI3Kγ, highly expressed in myeloid cells, has recently emerged as another key regulator of immunosuppressive macrophages (207, 208). In preclinical models, PI3Kγ selective targeting has been shown to reprogram macrophages into an immune-activating phenotype and to enhance ICIs activity (209). A Phase I study of the oral PI3Kγ inhibitor IPI-549 in combination with Nivolumab showed favorable tolerability and early signs of clinical activity in solid tumors. Upregulation of PD-L1 and CXCL9/10 and re-invigoration of exhausted PD1^+^CD8^+^CD45RA^−^ T cells were observed in blood samples during treatment, suggestive of immune activation and reduced immunosuppression (161). Even if no data are available for the modulation of myeloid cells in these patients, these encouraging results show that PI3Kγ inhibition might help overcome resistance to ICIs and have led to Phase II IPI-549 combinations with Nivolumab or Atezolizumab (Table 1).

### CD40 Agonists

As discussed previously and elsewhere (4, 5), myeloid cells can also have an anti-tumoral role through antigen-presentation and effector functions. The costimulatory protein CD40 is expressed by myeloid cells and DCs and, when activated by its ligand, promotes antigen presentation (210). A strong correlation between survival of CRC patients and CD40 expression in tumors was previously uncovered (211). In murine pancreatic tumor models, CD40 agonists were combined with anti-PD-1 and chemotherapy to trigger effective T cell immunity (212, 213). In a CRC model, a CD40 agonist led to PD-L1 increase on tumor-infiltrating monocytes and TAMs, PD-1 upregulation on T cells and a synergistic tumor growth inhibition in combination with an anti-PD-1 (214). Based on this preclinical evidence, the combination of the APX005M CD40 agonist with Nivolumab plus standard gemcitabine and nab-paclitaxel is currently being tested with promising antitumor activity in pancreatic cancer, where ICIs have been ineffective as single agents (215). In these patients, baseline biopsies revealed a low CD8^+^ T cell and a high macrophage infiltration. Moreover, the immune-profiling of PBMCs showed a rapid activation of dendritic cells in most patients upon treatment (162).

### STING Agonists

Type I interferon pathway is crucial in linking the innate and adaptive immune responses to mediate tumor rejection in mice and humans (216, 217). The activation of the STimulator of INterferon Genes (STING) pathway increases IFN-β production by tumor-resident DCs and induces the recruitment and priming of T cells against tumor antigens (218). The discovery of agonists of STING in mice [5,6-dimethyllxanthenone-4-acetic acid or DMXAA (219, 220)] and humans [MIW815/ADU-S100 and MK-1454 synthetic cyclic dinucleotides or small molecules like GSK3745417 (221, 222)] extended the possibilities of rational combinations with ICIs. Until the development of small-molecules suitable for systemic administration (223), clinical trials with the first STING agonists were focused on intratumoral delivery and thus limited to patients with accessible tumors.

In preclinical models, DMXAA, previously known for its antivascular properties (224) was shown to indirectly affect the release of TNFα and nitric oxide by TAMs (225, 226) and to induce the repolarization of M2-like into M1-like macrophages (227). DMXAA was able to promote rejection of B16 melanoma cells with an increased influx of CD8^+^ TILs (228) and triggered the cooperation between lymphocytes and monocytes, macrophages and neutrophils in murine breast cancer (229). However, due to distinct amino acids, DMXAA does not activate the human STING (219, 220), as confirmed by the negative results of Phase 3 trial in NSCLC patients (230). Several agonists specific for human STING have since been developed and recently entered the clinic. The combination of intratumoral MK-1454 plus Pembrolizumab resulted in encouraging efficacy and an acceptable safety profile in solid tumors or lymphomas (163). Moreover, the well-tolerated combination of intratumoral MIW815/ADU-S100 with the anti-PD-1 Spartalizumab has demonstrated antitumor activity in breast cancer and relapsed melanoma (164). MIW815/ADU-S100 is also being investigated in combination with anti-CTLA-4 in a Phase I trial. These STING agonists have demonstrated evidence of myeloid cell activation in patients through the induction of IL-6, CCL2 and type I IFN in the bloodstream and PD-L1 upregulation in tumors (231).

### All-Trans Retinoic Acid (ATRA)

One of the first molecules that has shown an effect on myeloid cells is ATRA, a vitamin A derivative that binds to the retinoic acid receptor on MDSCs and immature monocytes, differentiating them into mature DCs (232). This molecule is a standard treatment for patients with acute promyelocytic leukemia (233) but it has been tested in clinical trials for other indications, such as small-cell lung cancer, where anti-tumor immune responses where accompanied by a decrease in circulating total MDSC (Lin^−^CD33^+^HLA-DR^−^) and M-MDSCs (234). In a small clinical trial, melanoma patients treated with the combination of Ipilimumab and ATRA had significantly decreased circulating MDSCs when compared to Ipilimumab alone. Additionally, while a decrease in MDSCs was observed with the combination, the frequency of MDSCs increased over time in patients treated with Ipilimumab alone. Interestingly, compared to Ipilimumab alone, the combination induced an increased in circulating HLA-DR^+^ myeloid cells over time, accompanied by a significant decrease in eosinophils. The combination treatment was also associated with improved CD8^+^ T cell responses and the frequency of activated lymphocytes inversely correlated with that of circulating MDSCs in all patients (165). Even though patient enrollment in this study was halted following the approval of anti-PD-1 antibodies, the NCT03200847 clinical trial was launched with the aim of testing the combination of ATRA and Pembrolizumab, with an estimated completion date in June 2020.

### Trabectedin

Another myeloid-targeting agent that could improve the efficacy of ICIs is Trabectedin, a DNA-binder of marine origin approved as a single agent for the treatment of soft tissue sarcoma and, in combination with doxorubicin, for relapsed platinum-sensitive ovarian cancer (235, 236). Trabectedin not only directly kills tumor cells by interfering with cell cycle progression, but also modulates the TME via a selective depletion of TAMs and MDSCs (237). In a murine ovarian cancer model, the combination of Trabectedin with anti-PD-1 significantly prolonged mice survival, with a concomitant decrease in MDSCs and TAMs and a significant increase of effector CD4^+^FoxP3^−^ T cells and CD8^+^ T cells (238). Based on this evidence, several combination trials of Trabectedin and ICIs have been launched but the efficacy in patients is still undefined (Table 1).

### Anti-angiogenic Molecules

Myeloid cells in tumors can also be indirectly affected by drugs that are not specifically design to target them. As an example, the vascular endothelial growth factor (VEGF), in addition to its role in angiogenesis, has profound effects on immune cell functions: it inhibits DC maturation, antigen presentation and lymphocyte infiltration, while promoting Treg and MDSC expansion in the TME (239–243). Preclinical models and phase 1 studies suggest that anti-VEGF molecules might enhance the antitumor activity of ICIs by improving T cell infiltration, upregulating MHC I expression and reversing myeloid immunosuppression (244).

Based on this rationale, several clinical trials combining ICIs and antiangiogenic agents are currently ongoing (Table 1). The potential synergy of Ipilimumab and the anti-VEGF Bevacizumab (Ipi-bev) has been investigated in metastatic melanoma. Compared with pre-treatment or with post-treatment samples from the Ipilimumab group, the combination enhanced the intratumoral endothelial activation, resulting in increased trafficking of CD8^+^ T cells and CD163^+^ dendritic macrophages across the tumor vasculature. Although not functionally characterized, macrophages displayed extensive dendritic processes, suggesting that Bevacizumab might have increased their maturation and antigen-presenting capacity (177).

In the same trial, the authors found that high circulating Angiopoietin-2 (ANGPT2) [a vessel-destabilizing molecule and critical regulator of blood vessel maturation (74)] levels at baseline and early during treatment were associated with shortened OS and reduced response. Treatment with PD-1 blockade or Ipilimumab alone increased, whereas Ipi-Bev decreased, serum ANGPT2 in a significant proportion of patients (178). ANGPT2 binds to the Tie2 receptor and can have an impact on monocytes and macrophages subsets that express it (245–247). Tumor biopsies with high tumor vascular ANGPT2 expression showed an increase in CD68^+^ and CD163^+^ macrophages after Ipilimumab or Ipi-bev treatment. Ipi-bev treatment, however, decreased tumor vascular ANGPT2 expression in a subset of patients, together with a decreased CD68^+^ and CD163^+^ macrophage infiltration, suggesting that ANGPT2 might have a role in resistance to ICI through TAM recruitment and that Bevacizumab might influence myeloid infiltration also by acting on the ANGPT2 levels. Moreover, ANGPT2 promoted PD-L1 expression on M2-polarized macrophages *in vitro*, hinting at another potential mechanism of resistance in ICI-treated patients with increased amounts of ANGPT2. In conclusion, ANGPT2 might serve as a potential predictive biomarker for ICIs and a possible target for combinations that could help reduce myeloid cell infiltration and tumor immunosuppression (178). As a consequence, the ANGPT2 inhibitor Trebananib is currently being tested in combination with Pembrolizumab (Table 1) (248).

Several clinical trials combining ICIs with Bevacizumab are also ongoing in mRCC (Table 1), in which elevated serum and tumor VEGF levels have been associated with poor survival (249). In a study combining Bevacizumab with Atezolizumab, the authors demonstrated the ability of Bevacizumab to induce a Th1 signature with chemokines involved in lymphocyte trafficking, tumor MHC I protein expression and infiltration of tumor-specific T cell clones. As reported for Ipi-bev combination (177), the combination of Atezolizumab and Bevacizumab reduced the presence of CD31^+^ blood vessels, especially of immature, unstable ones, with a widespread infiltration of immune cells. Notably, the on-treatment localization of CD68^+^CD163^+^, but not CD68^+^CD163^−^ macrophages, was observed adjacent to immature, but not mature, vessels. Nonetheless, the role and modulation of distinct TAM subsets during Bevacizumab treatment needs to be further explored to better understand the immune-related mechanisms of action of anti-angiogenic drugs in ICI combos (172). Moreover, the anti-tumor activity seen with the combination was associated with a further increase in CD8^+^ T cells and unique T cell clones in the tumor, supporting the evaluation of this combination in phase 2 and 3 trials in mRCC and in other tumor types.

As discussed in the previous paragraphs, the IMmotion150 study was the first randomized trial to investigate the clinical activity of Atezolizumab with or without Bevacizumab against the standard-of-care Sunitinib in mRCC. Sunitinib efficacy was enriched in highly angiogenic tumors, while the combination of Atezolizumab and Bevacizumab improved clinical benefit compared with Sunitinib in T_eff_^high^ tumors. Atezolizumab monotherapy was effective in tumors with pre-existing immunity and a relatively low expression of myeloid-associated genes, while the combination with Bevacizumab improved the clinical outcome in T_eff_^high^Myeloid^High^ patients, confirming the ability of Bevacizumab to overcome myeloid-mediated resistance in these tumors (104).

## Conclusions

The use of ICIs has greatly changed the survival of a substantial fraction of patients with cancer in the last years. However, the knowledge about the mechanisms of primary and acquired resistance is still limited. The exploration of biomarkers in clinical trials is essential to understand how the immune system and the TME of each patient influence the response to ICIs and thus how the therapy should be personalized.

In this review we have drawn attention to the impact of myeloid cells on ICI therapy, with a special focus on clinical data. The existing evidence supports the exploration and the formal validation of myeloid subsets in blood and tumor as both predictive and pharmacodynamic biomarkers and the use of myeloid-targeting agents as rational partners for ICI combinations. Even though most studies point to a regulatory role of cells of the monocyte/macrophage lineage, different subsets might be prevalent in different cancer types. Accordingly, multiparametric technologies (multicolor flow cytometry, mass-cytometry, multiplex immunofluorescence and bulk or scRNA sequencing) are crucial for the study of biomarkers, as they allow a more detailed characterization of the phenotype, function and localization of subsets that are more informative than the simple abundance of macro-populations detected with classical methods. At last, the encouraging data from clinical combinations of ICIs with myeloid-targeting drugs support the idea that controlling the expansion, recruitment and function of myeloid cells in tumors is crucial to extend the benefit of these immunotherapies to non-responding patients.

## Author Contributions

All authors contributed to the article and approved the submitted version.

## Conflict of Interest

EP is currently employed by Servier as a Translational scientist in the Center for Therapeutic Innovation in Oncology. The remaining authors declare that the research was conducted in the absence of any commercial or financial relationships that could be construed as a potential conflict of interest.
